# Cases and Clinical Observations in the Medical Wards of the Illinois General Hospital

**Published:** 1852-03

**Authors:** N. S. Davis


### ARTICLE IV.

*Cases and Clinical Observations in the Medical Wards of the Illinois General Hospital, under the charge of* N. S. DAVIS, M. D., and Prof. Path., Practice and Clinical Medicine.

Case 1st. Mr. ———, young man, sailor, aged about 20 years, native of Ireland, admitted into the Hospital.

Dec. 23d. Present appearance: Lies with his head and shoulders much elevated; face bloated and of a leaden color; lips purple; tongue clean but pale; respiration difficult, labored, somewhat asthmatic; cough frequent, accompanied by soreness and some pain in the left side, and the expectoration of a thick, brownish, mucous; the bowels moderately costive; urine scanty and high colored; pulse 100 per minute and easily compressed; great muscular debility. Percussion gave a pretty clear sound over the whole right side of the chest, and over the left, from the clavicle down to the inferior margin of the pectoral muscles,but decided dullness over the axillary and sub-axilary and posterior inferior regions of the left side. Auscultation revealed a loud and mixed ronchus over the whole upper and central portions of both lungs. It consisted of the course mucous ronchus mixed with numerous dry cooing and whistling or hissing sounds. Over the lower margin of the pectoral muscle of the left side, a finer sub-mucous räle was distinct, while in the axillary and posterior regions of the same side there was scarcely any pulmonary sounds, except close in the axilla where *tubular* respiration was well marked and plain.

After allowing each member of the Hospital class to listen to the morbid sounds enumerated, before stating the diagnosis the Professor of Clinical Medicine gave the following history of the case:

The patient stated that he had had severe attacks of Dyspnœa, soreness, and pain in his chest, every winter for three or four winters past; that his present sickness commenced about ten days since, but did not confine him wholly to his bed until about four days since, when the dyspnœa became so great as to threaten entire suffocation, accompanied by the swollen and livid appearance of the face and other symptoms that still remain. We cannot learn that the expectoration has been at any time bloody or very copious, but rather glairy and very tenaceous. It was remarked that the history of the case together with the present symptoms and physical signs, clearly pointed out a complication of disease of great extent.

In the first place, the extensive mucous ronchus intermixed with dry bronchial sounds, over the more resonant parts of the chest, and the constricted or asthmatic character of the breathing, indicate an extensive chronic bronchial inflammation, probably of long standing and accompanied by thickening of the membrane, and consequent constriction or narrowing of the tubes. To this state of the bronchial membrane much of the dyspnœa, and livid aspect of the patient is due.

Second, the decided dulness over the lower and posterior portion of the left side, with some degree of sub-mucous ronchus especially over the margin of the dulness, and without fulness of the intercostal spaces or enlargement of the side, indicate a decided density of the pulmonary tissue of that region, from some cause. We may have unusual density of the pulmonary tissue from Pneumonia constituting hepatization; from œdema or engorgement of the tissue more passive than that constituting true hepatization, or from *carnification* by which is meant the conversion of the naturally loose and extremely vascular tissue of the lungs into a more dense and fibrous structure, as sometimes results from a species of chronic pneumonia. That the density in this case results from one or the other, or both of the latter conditions, is rendered probable from the absense of either bloody or purulent expectoration, which would almost certainly be present at this stage of active Pneumonia.

If we suppose the patient to have been long laboring under a slow bronchial inflammation, complicated at times during the two or three winters past, by congestions and low grades of inflammatory action in the middle and lower lobes of the left lung, we should be very likely to have that condensation or carnification of the pulmonary tissue of which we have spoken.

This would also account for the distinct tubular respiration high up in the axilla. If such a condition of the middle lobe of the lung had existed as the result of previous attacks of disease, the recent renewal of the extensive bronchial affection, and consequent dyspnœa which now exists, by interfering with the decarbonization of the blood, lowering the tonicity of the system and obstructing the pulmonary circulation, would be very likely to induce in the lower and posterior or most dependent part of the lungs an engorgement of the tissue sufficient to explain all the dulness existing in the present case, with only a very low grade of pneumonic inflammation. If these views of the nature of the case are correct, the indications for treatment are

1st. To relieve the respiration as much as possible by lessening the constriction of the bronchial tubes and facilitating expectoration.

2nd. To aid in relieving the blood of its excess of carbon resulting from the obstructed respiration, by increasing the secretions from the Liver and mucous folicles of the intestines.

3d. To remove the engorgement and condensation of the pulmonary structure, by improving general toncity and promoting absorption.

To fulfill these indications as far as possible, the patient was directed to take a tea-spoonful of the following mixture every four hours, viz:

℞. Sat. Tinct. Cimicifuga Racemosa, ℥jj,

Tinct. Lobelia Inflata, ℥ss,

Iodide Potassa, ʒj, mix.

In addition to this he was directed five grains of Blue Mass, to be followed by a table-spoonful of Castor Oil in the morning. Also a blister over the left axillary region.

Dec. 24th., 9 o'clock, A. M. The dyspnœa is much relieved, the lips consequently less livid; patient lies with his head and shoulders lower, and complains of less oppression and tightness across the chest. Otherwise the same as yesterday. Continue the same treatment.

Dec. 26th, 9 o'clock A. M. Patient much improved; breathing comparatively easy, pulse slower and more natural; bowels open; appetite moderate; cough less frequent, but considerable mucous expectoration, and occasional slight epistaxis or hemorrhage from the nose. He is able to take an entirely recumbent posture but pretty constantly inclines to the left side. The bronchial ronchi have much diminished, but not ceased. The dulness over the lower and posterior part of the left side, and the tubular respiration in the axilla remain unchanged. There is some œdema of the feet and ankles.

Omit the Tinct. Cimicifuga Racemosa and Iodide of Potassa and give Tinct. Sanguinaria Canadensis and Tinct. Lobelia, equal parts 30 gtts., every four hours, with five grains of Blue Mass at bed-time.

Dec. 29th, 9 o'clock A. M. Patient has continued to improve and is now able to sit up some, although the physical signs derived from an examination of the chest remain nearly the same. The ankles continue œdematous and the cough though less frequent is sometimes severe. The epistaxis is also sufficiently frequent and severe to be a source of debility

Continue the cough mixture last prescribed every six hours, with 10 grs. of Chloride of Sodium (common Salt) between each dose of the drops. The latter was prescribed for the purpose of counteracting the evident tendency to impairment of the qualities of the corpuscles and other constituents of the blood, and to improve the tonicity of the vascular tissues.

From this time forward he continued to improve steadily in breathing, strength, cough, &c. The dullness, however, continued well marked on the lower part of the left side, and the expectoration continued considerable presenting an appearance of slight intermixture of pus. On the 1st of January a blister was again applied to his side, otherwise the same treatment continued.

Jan. 3d. Finding considerable tendency to œdema of the lower extremities whenever the patient sits up much, with a cool skin, and free perspiration, two grains of the Sulphate of Quinine were added to each dose of the Chloride of Sodium, and the treatment otherwise continued the same.

Jan. 9th. The patient has continued apparently to improve slowly. On the 7th he walked out the distance of three or four blocks to see his friends and back again; and with the exception of some cough at night, inability to lie upon his right side, and œdematous swelling of the feet and ankles, he declared himself quite well. Still the dulness on percussion, the tubular respiration in the axilla, remained, but the bronchial ronchi had mostly disappeared. This morning he rose from his bed as usual, dressed himself, walked about the ward, but soon began to complain of a sense of suffocation, his breathing became rapidly laborious, his lips livid, his extremities cold, skin covered with a cold sweat, and just as the Prof. of Clinical Medicine entered the ward for the regular morning visit, he expired, apparently from suffocation. The whole period from the attack in the morning until death was little more than one hour.

*Post Mortem:* Twelve hours after death. Outward inspection. The chest and abdomen full and the intercostal spaces prominent at the upper part of the chest; emaciation moderate, and slight œdema of the lower extremities.

On opening the chest by raising the sternum, the upper lobes of both lungs presented themselves very strongly distended with air, constituting a true and extensive emphysema, which explained clearly the sudden death. The mucous membrane of the larger bronchial tubes on both sides was found injected, thickened and in places ulcerated. The tissue of the right lung was otherwise little altered from a healthy condition. On the left side, old and tough pleuritic adhesions existed to such an extent as to make it difficult to remove the middle and lower lobes from the chest. The whole lower lobe of the left lung was dense, heavy, inelastic, dark red color, and when cut into, was found in a semi-hepatized condition with here and there points of suppuration established. The central portion of the left lung, that which corresponded with the axillary region, was very closely adherent to the walls of the chest, and had been converted into a dense whitish, inelastic tissue, destitute of crepitant feel, or any appearance of infiltration. It was only slightly permeable to air, and was plainly the effect of previous disease. It was doubtless the density of this altered tissue with its close adhesion to the walls of the chest, that brought the larger central bronchial branch so near the ear as to give the plain tubular respiration heard in the axilla before death.

The heart was found moderately hypertrophied, with half an ounce or ounce of serum in the pericardium. The valvular strictures of the heart were perfectly natural, but both ventricles were filled with a firm clot of fibrin, from which the serum and red-corpuscles had been removed. In the left ventricle especially, the fibrinous clot was large, very white, and so firm that it might almost be stretched out into laminæ like a sheet of membrane. The formation of these clots, the nature of the morbid changes which had taken place in the lungs, the correspondence between the morbid appearances and the indications furnished by the symptoms and physical signs during life, and the extent and sudden occurrence of the emphysema, were fully explained to the Hospital Class present. But these are points that will appear too obvious to the reader, to need detail here.

*Case 2d.* Mr. M———, aged 35 years, native of Ireland, habits intemperate, admitted to the Hospital Jan. 6th, but was not seen by the Prof. of Clinical Medicine or the class until the regular hour for Hospital visit on the morning of the 7th. The attention of the class was first called to the position and aspect of the patient. He was bolstered up so as to assume nearly a sitting posture; his face pale with an anxious expression; his breathing short and rather hurried; his feet and ankles œdematous; with considerable swelling and tenderness of all the articulations from the second joint of the fingers to the shoulders, and from the lumbar region downward to the knees. The wrists and back of the hands were also œdematous, pitting freely on pressure. His pulse was 110 per minute, tongue slightly coated, no appetite, frequent distressing cough, paroxysms of great oppression and distress in the region of the heart; bowels inactive, and entire inability to move, with severe pains in the back, hips, arms, hands, and chest. The patient stated that he had been sick with Inflammatory Rheumatism about ten days, and had been directed by his physician to drink freely of sour milk and soda. The urine was scanty and high colored, but gave no traces of the presence of albumen. It was very evident, remarked Prof. D., that the patient has been laboring under the influence of severe Rheumatic Inflammation for several days, and which still continues to affect many important articulation. But this is not all. The tendency of the patient to keep the sitting posture or even to lean forward, the short respiration and cough, the paroxysms of severe oppression in the cardiac region, with the tendency to general anasarca, point very strongly to a pericardial or endocardial complication. On applying percussion, the cardiac dulness was found to occupy a larger space than natural; and auscultation revealed over the base of the heart, both a plain rubbing or friction sound, and a bellows murmur that almost entirely obscured the second sound of the heart. A degree of mucous ronchus indicating considerable bronchial inflammation was heard over the upper part of the left lung. After permitting each member of the Hospital Class to listen to these morbid sounds for himself, it was remarked that they pointed out plainly and unequivocally what we might suspect merely from the general symptoms, viz: the existence of Pericardial and Endocardial inflammation. This species of cardiac inflammation is by no means uncommon in connection with rheumatism of an acute and sub-acute grade; a fact which should always be borne in mind by the practitioner when treating the latter disease, as a failure to detect it, may lead either to an unexpectedly fatal result, or to such alterations of the membrane lining the heart and constituting the cardiac valves, as to constitute permanent organic disease.

*Prognosis:* Acute and sub-acute articular rheumatism very rarely terminates fatally. And even when complicated with Pericarditis or Edocarditis, if rightly understood and properly treated in the early stage, the prognosis is generally favorable. In patients of intemperate habits, however, like the one before us, or where the proper treatment has not been applied in the early stage, life may be cut short by the rapidity and extent of the pericardial effusion, or by the softening or impairment of the substance of the heart; or what is more common the valves and internal lining of the heart become permanently thickened, slowly producing hypertrophy and other incurable organic changes.

*Treatment:* In this case, the period when a free bleeding followed rapidly by full doses of Calomel and Opium, would speedily subdue both the rheumatic and cardiac inflammations, has passed by. The pale and œdematious appearance of the patient, the small and frequent pulse, and the intemperate habits all forbid any abstraction of blood. The indications now are, to allay the excessive irritability by sedatives, and arrest as speedily as possible the pericardial and dropsical effusions which have already begun, by promoting absorption and diuresis. To accomplish these objects the patient was directed to take the following, viz:

℞ Sat. Tinct. Cimicifuga Racemosa, ℥jj,
Iodide Potassa, ʒj, mix: one tea-spoonful every four hours; also, Pulv. Doveri, 10 grs., and Blue Mass, 5 grs. at bed-time, with a blister over the cardiac region.

Jan. 8th, 9 o'clock, A. M. Patient suffers less pain, both in the chest and extremities, otherwise symptoms not materially altered. Continue same treatment except the powder at bed-time, which was made to contain Pulv. Doveri, 10 grs., Nit. Potassa, 15 grs., and Calomel, 2 grs.

Jan. 11th. The treatment mentioned on the 8th, has been steadily continued, with a well marked beneficial effect. The œdema of the extremities has disappeared; the pain and swelling of the articulations have very much abated; the pulse is slower, but the breathing is still short with considerable cough and expectoration. Percussion also gives well marked cardiac dullness over too large a space, but the bellows murmur and the friction sound have nearly disappeared. In their place, however, we have less cardiac impulse, and the sounds of the heart apparently more remote from the ear, indicating the existence of considerable pericardial effusion. The patient is pale, complains much of debility, and the pulse is feeble. Discontinue the Iodide of Potassa and Tinct. of Cimicifuga, and give Citrate of Potassa, 15 grs., with Pulv. Doveri. 10 grs., every six hours.

Jan. 14th. Symptoms of cardiac disease almost entirely removed, and the appearance of external rheumatism slight. But there is pretty copious expectoration, much debility, with periods of profuse sweets. The bowels were also too loose and the evacuations watery. Ordered three grains of Sulph. Quinine to be added to each of the powders as previously taken, and the bowels to be restrained by the following emulsion, viz:

℞ Oil Turpentine, ʒjj.,
Tinct. Opii., ʒjj.,
Gum Arabac and White Sugar, each ʒiij., rubbed thoroughly together with water ℥ij., of which a tea-spoonful may be given as often as the bowels move.

Jan. 17th. The evacuations continue watery and fœtid, abdomen moderately tympanitic, and an increase of the external rheumatic pains. The pulse was also more frequent and irritable, and more bronchial irritation and cough. The physical signs indicated no increase of cardiac inflammation, and the extent of dullness was not much greater than natural.

The increase of the bronchial and rheumatic irritation appeared to be produced by the Quinine acting on a bad condition of the bowels. All previous medicine was discontinued and the patient directed the following powder every four hours, viz:

℞ Pulv. Doveri., 10 grs.,
Nit. Potassa., 10 grs.,
Calomel, 2 grs., mix; with a blister again applied to the left side of the chest.

Jan. 18th. Discharges from the bowels much changed, abdomen less tympanitic, breathing and cough easier. Omit the powders with Calomel, and order the following, viz·

℞ Citrate Potassa, 15 grs.,
Pulv. Opii., 1½ grs., mix. Give one powder morning, noon, and tea-time; with the following powder at bed-time, viz:

℞ Nit. Potassa, 15 grs.,

Pulv. Opii., ½ gr., mix.

Jan. 26th. Since the 18th, the patient has steadily improved. The cardiac and rheumatic symptoms have entirely disappeared. The patient, however, is anemic, somewhat emaciated, and still coughs and expectorates considerable opake or semi-purulent matter. While making the free use of Opium as prescribed on the 18th, his bowels required moving occasionally with Castor Oil to which was usually added Oil of Turpentine. A third blister was also applied over the left subclavicular region on the 23d inst. The patient was now ordered, simply a table-spoonful of Cod-Liver Oil with ten drops of Acetum Opii, three times a day with nutritious diet. Under this he improved rapidly, and at the present time, Feb. 10th, is quite well and walking about the ward.

